# Immunogenic Peptides from Pap31 and SCS-α of *Bartonella bacilliformis*: One Step Closer to a Rapid Diagnostic Tool for Carrion’s Disease

**DOI:** 10.3390/pathogens10080917

**Published:** 2021-07-21

**Authors:** Cláudia Gomes, Maria J. Pons, Juana del Valle-Mendoza, Mayumi Matsuoka, Joaquim Ruiz

**Affiliations:** 1 ISGlobal, Hospital Clínic—Universitat de Barcelona, 08036 Barcelona, Spain; 2 Laboratorio de Microbiología Molecular y Genómica Bacteriana, Universidad Científica del Sur, Lima 15067, Peru; ma.pons.cas@gmail.com; 3 School of Medicine, Research and Innovation Center of the Faculty of Health Sciences, Universidad Peruana de Ciencias Aplicadas (UPC), Lima 15023, Peru; juanadelvalle@upc.com; 4 Laboratorio de Biologia Molecular, Instituto de Investigación Nutricional, Lima 15024, Peru; 5 National Institute of Infectious Diseases, Tokyo 208-0011, Japan; kubotama@nih.go.jp

**Keywords:** Carrion’s disease, *Bartonella bacilliformis*: diagnostic, immunogenic tools, peptides, ELISA

## Abstract

*Bartonella bacilliformis* is the causal agent of Carrion’s disease, an overlooked illness endemic in the Andean Mountains with Peru being the most affected country. The diagnostic of this illness is a challenge due to the limited resources and the common symptomatology with other infectious diseases. The goal of this study was to identify immunogenic peptides from Pap31 and succinyl-CoA synthetase α (SCS-α) of *B. bacilliformis* that might be suitable for developing a serologic tool. The immunodominant character of Pap31 and SCS-α was determined by Western blotting and in-silico analysis. Subsequently, 35 peptides were selected for epitope mapping and their immunoreactivity was tested by enzyme-linked immunosorbent assay (ELISA). A total of 30 sera were tested including pre-exposed people with high IgM levels for Pap31/SCS-α (23 sera), patients (2 sera) as well as 5 sera with no reactivity to Pap31/SCS-α. The results indicate that Pap31-8 (_187_QAIGSAILKGTKDTGT_202_) and SCS-α-12 (_59_IFASVAEGKEKTGANA_74_) are the most immunogenic peptides, with Pap31-8 showing potential to discriminate between *B. bacilliformis* and the remaining *Bartonella* spp., and SCS-α-12 differentiating *Bartonella* spp. from other microorganisms.

## 1. Introduction

*Bartonella bacilliformis* is the microorganism responsible for Carrion’s disease, an overlooked illness restricted to the Andean region of Peru, Ecuador, and Colombia [1]. This illness is transmitted by the bite of a sandfly, predominantly *Lutzomyia verrucarum,* and up to now, humans are the only established reservoir [1]. There are three well-defined clinical manifestations of this disease: the acute phase or Oroya fever in which *B. bacilliformis* invades the erythrocytes and include the following main symptoms: hemolytic anemia, malaise, fever, headache, and mild chills, as well as pallor, hepatomegaly, abdominal pain, and other unspecific symptoms [1,2], with lethality of up to 88% in the absence of treatment [3]. Meanwhile, in the chronic phase, *B. bacilliformis* produces an abnormal proliferation of endothelial cells causing characteristic dermal eruptions named Peruvian warts [1]. In this phase, mortality is considered irrelevant [1]. Finally, in the asymptomatic cases, the individual does not present any symptoms but is an asymptomatic carrier, thereby perpetuating the illness [4,5]. In the studies by Chamberlin et al., 0.5% of the population living in endemic areas presented asymptomatic bacteremia, and an indirect fluorescence antibody (IFA) assay showed that 45% of the volunteers were seropositive for *B. bacilliformis* antibodies [4,6]. Studies using more sensitive techniques, such as real-time PCR, detected approximately 40% of asymptomatic carriers in post-outbreak and endemic areas [5].

The diagnosis of Oroya fever is mainly based on clinical parameters and/or Giemsa-stained peripheral blood smears [2,7]. The common symptomatology with other infectious diseases present in endemic areas and the low sensitivity of around 30% with microscopy makes it extremely difficult to correctly diagnose acute patients [7,8,9,10,11].

The identification and evaluation of *B. bacilliformis* antigenic proteins would aid in the development of better diagnostic tests and could be a prelude to future testing of candidates for vaccine antigens. Succinyl-CoA synthetase α (SCS-α) and Pap31 are 2 of the main antigenic proteins of *B. bacilliformis* [5,12]. Pap31 is a 31.15 kDa-sized protein homolog of a bacteriophage-associated protein in *Bartonella henselae* (also called HbpA) and was first identified as an antigen of *B. bacilliformis* by immunoblotting with a patient serum [12]. An ELISA using the recombinant protein has already been described in the literature [13], and Pap31 was also considered a potential antigen candidate for a synthetic vaccine [14]. SCS-α is an essential protein of 30.99 kDa involved in the tricarboxylic acid cycle, which has been identified as an immunogen in studies developed in *Brucella* spp. [15,16]. Both Pap31 and SCS-α antigens were selected for this study because these two antigens were identified using an anti-human IgM as a secondary antibody [5], demonstrating their potential use for the identification of acute cases of Carrion’s disease, the phase of the illness with the highest mortality.

In order to avoid cross-reactivity among *B. bacilliformis* and other *Bartonella* species or other organisms, more specific and conserved antigens are essential as candidates for use in the serological diagnosis of this infection. Therefore, the objective of this work was to identify which peptides from the SCS-α and Pap31 antigens are the most immunogenic.

## 2. Results

**Figure 1:**
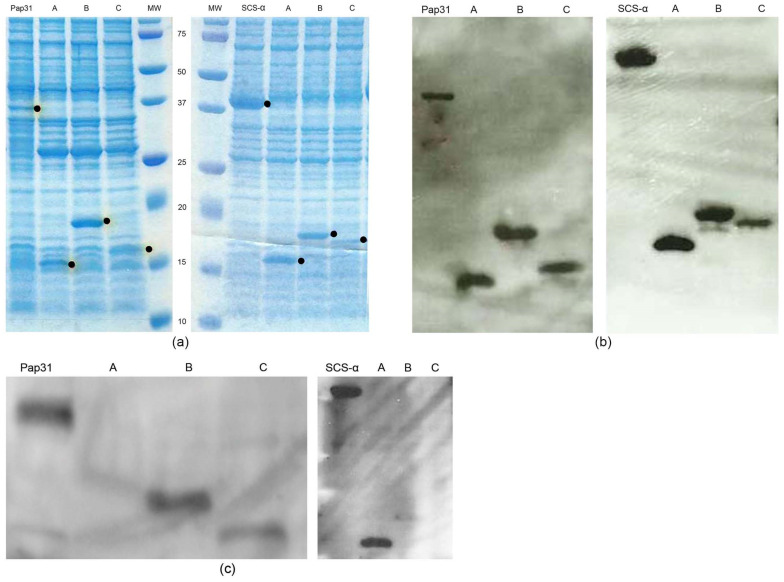
Expression and detection of Pap31 and SCS- α and their respective truncated constructs; SDS-PAGE showing the expression of the proteins of interest, full-length Pap31 and SCS- α and respective clones A, B, and C, corresponding to the three truncated constructs. The proteins of interest are marked with a dot. (**a**); Western blotting performed with the anti-Xpress antibody signalizing the proteins of interest. (**b**); Example of Western blotting performed with serum from exposed people for Pap31 and SCS-α and respective clones A, B, and C (**c**).

After cloning the complete proteins of Pap31 and SCS-α and their respective truncated constructs (Pap31–A, Pap31–B, Pap31–C, SCS α–A, SCS α–B and SCS α–C), we performed Western blotting showing that the immunogenic region of Pap31 antigen is contained in clones B and C, and for SCS-α, the immunogenic region is in clone A (Figure 1).

**Figure 2:**
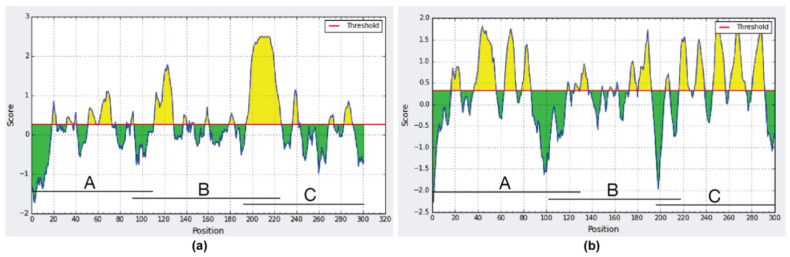
In-silico analysis of the immunogenic regions of (**a**) Pap31 protein; (**b**) SCS-α protein.

The in-silico analysis using the IEDB Analysis Resource software confirmed the immunogenic region for SCS-α was on clone A, while that of Pap31 was between clones B and C as shown in Figure 2. This in-silico analysis allowed the immunogenic zone to be analyzed to be reduced, and a series of overlapping peptides from amino acids 166 to 238 (Pap31) and 26 to 89 (SCS-α) was designed for further determinations.

**Figure 3:**
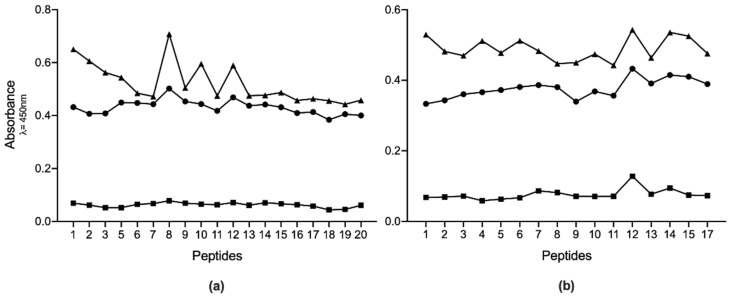
Absorbance levels of IgM ELISAs for all the antigenic peptides studied. (**a**) results for Pap31, and (**b**) results for SCS-α. Squares are the mean results for non-reactive samples; circles are the mean results of recently exposed sera, and triangles are the mean results for the positive samples.

The immunogenicity of the overlapping Pap31-B and Pap31-C regions, as well as SCSα–A, was tested by ELISA and the results are presented in Figure 3.

**Figure 4:**
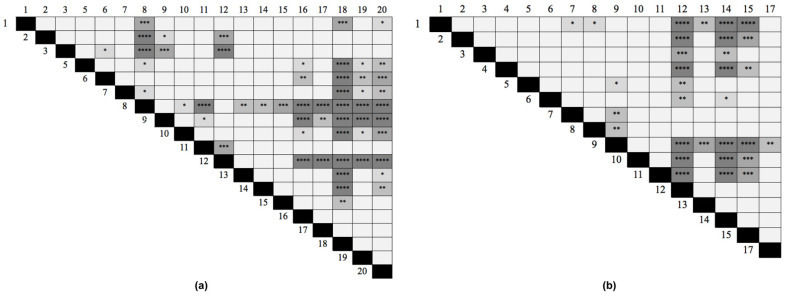
Differences in the immunogenicity among peptides calculated with the Friedmann test. (**a**) Pap31, (**b**) SCS-α. *p* < 0.0001 (****); *p* < 0.0005 (***); *p* < 0.005 (**); *p* < 0.05 (*).

Two different analyses were performed to establish which peptides could be the best candidates for use in a future diagnostic test. First, for sera from recently exposed people, we determined in which peptides the mean absorbance was higher than the remaining sera. The results showed that Pap31-8 (_187_QAIGSAILKGTKDTGT_202_) and SCS-α-12 (_59_IFASVAEGKEKTGANA_74_) presented higher reactivity values (Abs_450_ 0.502 and 0.433 respectively), followed by Pap31-12 and Pap31-9, and SCS-α-14 and SCS-α-15, respectively. The statistical analysis showed that Pap31-8 was significantly more immunogenic than all the Pap31 peptides, except for Pap31-12, Pap31-9 and Pap31-6. Meanwhile, SCS-α-12 was significantly more immunogenic than nine other SCS-α peptides (Figure 4).

**Figure 5:**
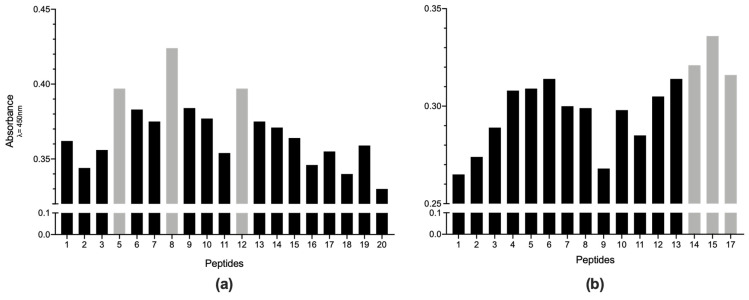
Immunogenicity levels for each peptide of (**a**) Pap31 and (**b**) SCS-α. The immunogenicity level of each peptide corresponds to the difference between the mean values of pre-exposed and non-reactive sera.

On the other hand, when we determined the peptides resulting in a higher difference between the mean values of pre-exposed and non-reactive sera, Pap31-8 was the most immunogenic Pap31 peptide, followed by Pap31-5 and Pap31-12 (both showing the same value) (Figure 5). These results confirm that the middle region of Pap31 is the most immunogenic, with Pap-31-B and Pap31-C overlapping. In this regard, in SCS-α, the peptide SCS-α-15 (_68_EKTGANASVIYVPPAG_83_) is the most immunogenic, followed by SCS-α-14 and SCS-α-17.

The in-silico specificity analysis was done for the peptides that showed higher immunogenicity in both analyses (Pap31-8 and SCS-α-12 in the Friedman test and SCS-α-15 when we compared the mean values of pre-exposed and non-reactive sera). The results showed that Pap31-8 was conserved in almost all the *B. bacilliformis* genomes sequenced, with the exception of strain Ver-097 (Reference Sequence: WP_041848867) which differed in 1 amino acid. No identity higher than 65% was found with the remaining *Bartonella* spp. genomes.

A 100% identity was found between SCS-α-12 and the corresponding region of the SCS-α protein of 6 *B. bacilliformis* genomes, with slight differences (1 amino acid) with the remaining 10 *B. bacilliformis* genomes present in GenBank. Similarly, SCS-α-15 was fully conserved in 15 out of 16 *B. bacilliformis* genomes present in GenBank, while in the remaining genome (strain Ver097, Reference Sequence: WP_041849026), 1 amino acid difference was observed. In contrast to Pap-31-8, both SCS-α-12 and SCS-α-15 peptides showed high identity with other members of the *Bartonella* genus, including all the human pathogenic members (either withstanding nomenclature or *Candidatus* species) present in GenBank.

In any case, we detected an identity higher than 90% with microorganisms belonging to other bacterial genera. Nevertheless, SCS-α-12 showed 87.50% identity with the corresponding region of the SCS-α protein of members of the genus *Brevundimonas* and 81.50% with *Brucella* spp. (Table 1). It is important to highlight that the peptide SCS-α-15 presented an identity higher than 80% with the SCS-α of several pathogenic microorganisms, including *Brucella* spp., such as *Brucella abortus* or *Brucella melitensis* (87.50%), or *Anaplasma* spp., such as *Anaplasma phagocytophilum*, *Brevundimonas* spp. such as *Brevundimonas diminuta*, *Enterobacteriales*, such as *Escherichia coli* or *Salmonella enterica*, different *Rickettsia* spp., such as *Rickettsia australis*, *Rickettsia prowazekii*, *Rickettsia rickettsii*, *Rickettsia sibirica* or *Rickettsia slovaca*, among others, with an identity of 81.25% (data not shown).

When the peptides were compared with the corresponding human protein, the highest identity observed was for SCS-α-12 and SCS-α-15 with 75% of identity due to four amino acid substitutions (Table 1 and data not shown).

## 3. Discussion

The development of robust diagnostic tools is essential for the control of infectious diseases. In the last years, the design of diagnostic tools has involved a series of high throughput molecular approaches, including matrix-assisted laser desorption/ionization-time of flight (MALDI-TOF) and next-generation sequencing, among others [20,21]. Nonetheless, these techniques remain economically and technically inaccessible in most low- and middle-income countries, especially in remote areas where the majority of Carrion’s disease cases occur, and the lack of communication in these areas, the limited resources, and non-adequately equipped laboratories can be added to this challenging scenario [1]. While Carrion’s disease may seriously compromise a patient’s life [1,3], the initial symptoms of the disease are similar to other illnesses present in the same areas [7], often leading to misdiagnosis [7,8,9]. Furthermore, a high percentage of asymptomatic carriers acting as a *B. bacilliformis* reservoir have been described [4,5]. In these conditions, the development of a low-cost, easy-to-use diagnostic tool, able to detect acute cases and also sensitive enough to detect asymptomatic carriers is essential for the control and elimination of Carrion’s disease.

Previously, we identified Pap31 and SCS-α as *B. bacilliformis* antigens that are strongly reactive to sera from pre-exposed people when using an anti-human IgM as a secondary antibody [5]. These results indicated that antibodies to these proteins are strongly induced by *B. bacilliformis* infection and are therefore potential candidates for developing a diagnostic tool for recent infections. Nonetheless, the use of short antigenic peptides versus whole proteins may result in enhanced specificity eliminating false positives caused by cross-reaction with other antibodies [22]. Previous studies highlighted the potentiality of peptides as candidates for the development of diagnostic tools in sera [22,23]. The most common antigens are from the outer membrane proteins and in particular for *Bartonella* spp. infections, the VompA, VompB, PpI, and hemin-biding proteins from *B. quintana* [24,25] and Pap31 and BH11510 from *B. henselae* [26] have been proposed as possible candidates.

Access to proteins for the development of immunogenic assays designed to detect specific immunogenic regions is limited and cumbersome, but in the current “omic” era, accessibility to directly sequenced proteins or inferred amino acid sequences from genomic DNA analysis is immense. The use of in-silico computer tools might allow predicting the most promising immunogenic regions based on standardized parameters in a fast and reliable manner, facilitating both the study of proteins independently of their physical availability and the selection of the protein regions to be analyzed. Thus, after selecting the putative immunogenic regions of Pap31 and SCS-α, we included short overlapping peptides covering the desired area in epitope mapping studies and identified Pap31-8 and SCS-α-12 as the most immunogenic peptides of these proteins.

In other *Bartonella* species, such as *B. quintana*, Pap31 binds to environmental heme [27], highlighting the presence of this protein on the bacterial surface. In addition to *B. bacilliformis*, an immunogenic role of Pap31 has been proposed in *B. henselae* [26]. In-silico analysis has revealed that the immunogenic region of *B. henselae* Pap31 is placed around amino acid positions 60–80 (data not shown), different from the *B. bacilliformis* immunogenic peptide Pap31-8. This should support the lack of cross-reactivity to *B. bacilliformis* Pap31-8 from patients infected with *B. henselae*. Indeed, in a study by Angkasekwinai et al., an ELISA performed with recombinant Pap31 from *B. bacilliformis* showed no cross-reactivity when testing samples containing antibodies against *Coxiella burnetti*, *Brucella* spp., and *B. henselae* [13]. Further analysis showed several differences in the surroundings of peptide Pap31-8 between *B. bacilliformis* and the remaining *Bartonella* spp., highlighting the presence of a repetitive region just after Pap31-8 [28].

Together with SCS-α-12, the neighboring SCS-α-14 and SCS-α-15 were the second and third most immunogenic peptides for SCS-α, highlighting the immunogenicity of this region (SCS-α-15 being the peptide showing the highest difference in reactivity compared to non-reactive sera). While no data has been found about the cytoplasmatic, membrane, periplasmatic, or secretory nature of SCS-α in *B. bacilliformis*, the immunogenicity of either SCS-α or SCS-β, the two subunits of Succinyl-CoA Synthetase, have been highlighted in different microorganisms, such as *Brucella* spp. or *Leptospira interrogans* [15,16,29]. Furthermore, it has been suggested that SCS-α is a relevant factor involved in *B. henselae* interactions with host cells [30].

The in-silico analysis showed that while the peptide SCS-α-12 and SCS-α-15 have identity levels between 81.25 and 93.75% with the equivalent region of SCS-α from other *Bartonella* spp. (as well as with specific isolates of *B. bacilliformis*), Pap31-8 is highly specific to *B. bacilliformis*, with no identity with any other *Bartonella* spp. higher than 65%. It was of note that the *B. bacilliformis* strain Ver097, which showed lower levels of identity for both Pap31 and SCS-α, has been considered as a possible *B. bacilliformis* subspecies or a phylogenetically related species [18,19]. Although further studies are needed to establish the cross-reactivity with other *Bartonella* spp. as well as other pathogens, this finding suggests that Pap31-8 might be a reliable candidate for detecting *B. bacilliformis* infections. Identity levels higher than 80% with SCS-α-15 of *B. bacilliformis* were found among numerous human pathogenic microorganisms, several of which were phylogenetically close, such as *Brucella* spp. or *Rickettsia* spp., and also more distant, such as *E. coli* or *S. enterica*, likely hindering the usefulness of this peptide for diagnostic purposes.

Two main limitations of this study need to be considered; (1) the limited number of sera used from patients with acute infections (nonetheless, the sera used from recently exposed people showed only a slight decrease in reactivity as compared to the patient samples), and; (2) the lack of sera from people with other infections (including other *Bartonella* spp.), which would definitively confirm the specificity of the selected peptides.

In summary, we describe three short peptides able to detect the presence of *B. bacilliformis* antibodies. While further studies are needed, at least one of these immunogenic peptides, Pap31-8, seems to be a strong candidate for the future development of a rapid diagnostic tool, which is essential to improve the control, elimination, and eradication of Carrion’s disease, a neglected infectious disease.

## 4. Materials and Methods

### 4.1. Serum Samples

In total, 46 serum samples were used in this study; the majority (44 samples) were obtained during a 2014 study conducted in 5 different villages in the Piura department (northern Peru). The villages consisted of 1 endemic and 4 post-outbreak areas of Carrion’s disease [5]. Thirty-six of the sera samples were from people with a positive ELISA result performed with the full-length proteins of Pap31 or SCS-α as antigens and anti-human IgM as a secondary antibody and were, therefore, considered to be from people who had recently been exposed. Eight sera from people living in the endemic area with a negative result for the same assay [5] were also included and considered as non-recently exposed (thereafter, referred to as non-reactive). Additionally, 2 samples from patients with the acute phase of the disease collected a few months previously in the same department were also included (positive samples).

### 4.2. Bacterial Strains

The microorganisms and plasmids used in this study are listed in Table 2. *B. bacilliformis* (CIP 57.20) was cultured at 28 °C on Columbia agar with 5% sheep blood for 7 days. The *E. coli* strains for cloning were grown on Luria-Bertani medium, supplemented with 50 µg/mL of ampicillin at convenience.

### 4.3. Amplification, Cloning, and Purification of Antigenic Proteins and Respective Truncated Constructs

In order to identify the immunogenic portion of Pap31 and SCS-α, 3 truncated constructs of 110–130 amino acids of Pap31 (Pap31-A: amino acid 1 to 108; Pap31-B: amino acid 92 to 225 and Pap31-C: amino acid 193 to 301) and SCS-α (SCSα-A: amino acid 1 to 127; SCSα-B: amino acid 101 to 217 and SCSα-C: amino acid 196 to 301) were created. Genes coding for the antigenic proteins as well as the respective truncated constructs were amplified by PCR using the primers listed in Table 3. Of note, the *pap31* and *sucD* genes of *B. bacilliformis* 57.20 were sequenced in a previous study, being identical to those belonging to reference strain KC583 [5].

Amplified products were purified using the MinElute PCR purification Kit (QIAGEN, Hilden, Germany) and cloned in Champion pET Directional 100/D-TOPO vector (Invitrogen, Carlsbad, CA, USA), according to the manufacturer’s instructions in order to generate Xpress-tagged versions of the candidate antigenic proteins. The DNA sequences were verified by Sanger sequencing using the following primers: M13F 5′-GTAAAACGACGGCCAG-3′ and M13R 5′-CAGGAAACAGCTATGAC-3′.

For the detection, *E. coli* isolates with the vector of interest were grown for 3 h in the presence of isopropyl ß-D-1-thiogalactopyranoside (IPTG) 1mM and thereafter suspended in lysis buffer (50 mM potassium phosphate, pH 7.8, 400 mM NaCl, 100 mM KCl, 10% glycerol, 0.5% Triton X-100, 10 mM imidazole). All proteins were separated on a 10–20% SDS-PAGE (FUJIFILM Wako Pure Chemicals Corporation, Osaka, Japan) and immunoblotted with anti-Xpress antibody (Invitrogen, Carlsbad, CA, USA). The complete proteins of Pap31 and SCS-α were previously purified using a His-Binding kit and following the manufacturer’s instructions (GE Healthcare, Buckinghamshire, UK) [5].

### 4.4. Western Blotting

Western blotting was performed to determine the immunogenic region of Pap31 and SCS-α. Both the purified complete proteins and their respective 3 truncated constructs were electrophoresed on 15% SDS-PAGE gels and electrotransferred onto a PVDF membrane (GE Healthcare, Buckinghamshire, UK) using a Bio-Rad Trans-Blot SD Cell apparatus. A blocking step for 1 h at room temperature using 5% skim milk in PBS was performed. The membranes were immunoblotted during 90 min with sera (1:50 dilution in Tris-buffered saline [TBS]-Tween with 1% bovine serum albumin [BSA]). Western blottings with at least 10 sera from exposed people were performed for both Pap31 and SCS-α. A commercial negative control (DAKO X0939) consisting of a pool of at least 30 sera from healthy blood donors was used. After washing 3 times with TBS-0.05% Tween20) for10 min, the membranes were incubated for 1 h with a polyclonal rabbit anti-human IgM 1:1000 dilution in TBS-T with 1% BSA conjugated with peroxidase (Dako, Glostrup, Denmark) using o-phenylenediamine tablets as substrate (Sigma, St. Louis, MO, USA).

### 4.5. In-Silico Analysis

An in-silico analysis using IEDB Analysis Resource software (http://tools.iedb.org/main/, accessed on 13 July 2021) was also performed. In addition to confirming the in vitro results, this in-silico analysis shortened the immunogenic zone of Pap31 and SCS-α.

### 4.6. Design and Synthesis of Biotinylated Peptides

A total of 35 overlapping peptides (Table 4) were purchased from GL Biochem Ltd. (Shanghai, China) corresponding to the immunogenic regions identified, 19 peptides corresponding to Pap31, and 16 peptides to SCS-α. The peptides were designed by Epitope Mapping, with a size of 16 amino acids each, offset by 3 amino acids relative to the former, 13 of which are therefore coincident between consecutive peptides. A molecule of biotin was coupled at the N-terminus of each peptide in order to obtain more signals when performing the ELISA technique. Peptide purity was quantified by the manufacturer, being at least 95%. The peptides were dissolved according to the instructions provided by GL Biochem and stored at −80 °C until use.

### 4.7. ELISA

A total of 30 sera were selected to be tested with each peptide. Of these, 23 were from pre-exposed people with high IgM levels for Pap31 or SCS-α (accordingly to the peptide to be tested), 2 from patients with Carrión’s disease, and 5 that had no reactivity to Pap31 or SCS-α. To determine the immunogenicity of each peptide, ELISA tests were carried out using the synthetic biotinylated peptides as an antigen. Briefly, 96-well Nunc-Immuno Maxisorp microtiter plates (Nalgene Nunc International, Roskilde, Denmark) were coated with 100 μL (0.67 μg/well) of NeutrAvidin biotin-binding protein (Invitrogen, Carlsbad, CA, USA) overnight at 4 °C. After washing with phosphate-buffered saline (PBS) 0.1% Tween-20, 100 μL of a single biotinylated peptide (28.6 μg/mL in each well) was added and incubated for 1 h. The plates were washed 3 times, and sera diluted 1:100 with TBS-Tween with 1% BSA were incubated for 2 h. The same negative control and secondary antibody used in the Western blottings were used here. The optical densities were measured as absorbance at 450 nm after using o-phenylenediamine tablets as substrate (Sigma, St. Louis, MO, USA). Each sample was analyzed in triplicate intra-plate.

### 4.8. Selection of the Best Immunogenic Candidates

The best immunogenic candidates were selected following two criteria of analysis. Thus, the means of the absorbance values obtained in the ELISA of the sera of recently-exposed people as well as the difference between the mean values of all pre-exposed and all non-reactive sera were determined. Peptides showing the highest mean absorbance when pre-exposed sera were tested and those presenting higher reactivity differences when pre-exposed sera and non-pre-exposed sera were compared were considered.

The specificity of the selected antigenic peptides was determined by comparison with GenBank. The identity levels were determined by comparing the peptide sequence with protein sequences of *Bartonellaceae*, bacterial pathogens, and humans.

### 4.9. Statistical Analysis

The statistical analysis was performed using GraphPad Prism. Normality of the data was accessed by the Shapiro test and the mean reactivity of each peptide was compared with the mean reactivity of every other peptide using the Friedman test. Significance was considered with *p* < 0.05.

## Figures and Tables

**Table 1:** Amino acid differences observed in the specificity analysis of the selected peptides.

Peptide	% ^1^	Organism ^2^	Protein ^3^	GenBank ^4^	Sequence
QAIGSAILKGTKDTGT	100.00	***Bartonella bacilliformis*^5^**	Pap31	WP_005767899	QAIGSAILKGTKDTGT
	93.75	***Bartonella bacilliformis*^6^**	Pap31	WP_041848867	QAIGSAILKGVKDTGT
	68.75	*Escherichia coli*	MdtK	EEW1185817	QAIGSGILRGYKDTRS
	68.75	*Klebsiella pneumoniae* ^7^	MdtK	WP_032418890	QAIGSGILRGYKDTRS
IFASVAEGKEKTGANA ^8^	100.00	***Bartonella bacilliformis*^9^**	SCS-α	WP_035454724	IFASVAEGKEKTGANA
	93.75	***Bartonella bacilliformis*^10^**	SCS-α	WP_005765714	IFASVTEGKEKTGANA
	93.75	***Bartonella bacilliformis*^6^**	SCS-α	WP_041849026	IFASVAEGKEKTEANA
	93.75	*Bartonella ancashesis* ^10^	SCS-α	WP_053943861	IFASVMEGKEKTGANA
	93.75	*Bartonella alsatica*	SCS-α	WP_005866529	IFASVAEGKEKTGADA
	93.75	*Bartonella clarridgeiae*	SCS-α	WP_013545653	IFASVVEGKEKTGANA
	93.75	*Bartonella henselae*	SCS-α	WP_005866529	IFASVAEGKEKTGADA
	93.75	*Candidatus* Bartonella melophagi	SCS-α	WP_007476332	IFASVAEGKEKTGADA
	93.75	*Bartonella schoenbuchensis*	SCS-α	WP_010704443	IFASVAEGKEKTGADA
	93.75	*Bartonella vinsonii*	SCS-α	WP_010705938	IFASVAEGKEKTGADA
	87.50	*Bartonella elizabethae*	SCS-α	WP_005774305	IFASVAEAKEKTGADA
	87.50	*Bartonella koehlearae*	SCS-α	WP_034458042	IFASVAEGKEKTDADA
	87.50	*Bartonella quintana*	SCS-α	WP_011179955	IFASVAEGKERTGADA
	87.50	*Bartonella rochalimae*	SCS-α	WP_035006433	IFASVVEGKEKTGADA
	87.50	*Candidatus* Bartonella tamiae	SCS-α	WP_008037783	IFSSVAEGKEKTGADA
	87.50	*Bartonella tribocorum*	SCS-α	WP_100129872	IFASVAEAKEKTGADA
	87.50	*Candidatus* Bartonella washoensis	SCS-α	WP_006925899	IFSSVAEGKEKTGADA
	87.50	*Brevundimonas* spp.	SCS-α	WP_087141820	IFASVAEGKERTGADA
	81.25	*Bartonella grahamii*	SCS-α	WP_015857104	IFANVAEAKEKTGADA
	81.25	*Bartonella doshiae*	SCS-α	WP_004856238	IFASVAEAKEKTDADA
	81.25	*Brucella* spp.	SCS-α	WP_004684457	IFATVAEGKERTGADA
	68.75	*Nocardia* spp.	SCS-α	WP_067485390	VFASVAEAMEKTGADT
	75.00	Human	SCS-α	SJM31584	IFSTVAEGKATTGANA

Amino acid differences are highlighted in bold and underlined. ^1^ %: Percentage of identity. Percentages below 65% were not considered. SCS-α-15 is not in the table due to the high number of identities accomplishing these criteria. ^2^ Only human pathogens. ^3^ When more than one variant of the same protein is present in GenBank only the one with the highest identity is reported. When two or more proteins have the same level of identity only one is stated. ^4^ When more than one amino acid sequence is reported for the same species or when microorganisms belonging to the same genus (other than *Bartonella* spp.) possess an identical protein, only one representative sequence is reported. The sequences starting with “WP” are all non-redundant sequences classified as “Reference Sequences” in GenBank. Additional information may be found at “https://www.ncbi.nlm.nih.gov/refseq/about/nonredundantproteins/ accessed on 13 July, 2021”. ^5^ All but one GenBank recorded *B. bacilliformis* sequences (either partial or whole genomes). ^6^ Strain Ver097. Note that this strain has been qualified as a group outlier by MLST studies [17]. In fact, it has been considered as a *B. bacilliformis* subspecies or a misidentified species related to *B. bacilliformis* [18,19]. ^7^ The related MdtK proteins have been described in other pathogenic microorganisms within *Enterobacteriaceae*, either belonging to the *Escherichia* or *Klebsiella* genus or not (e.g., *Salmonella*, *Shigella*, or *Citrobacter*, among others) but the identity was less than or equal to 62.5%. Only the non-pathogenic *Candidatus* Purcelliella pentastirinorum (AXN02026) possess an MdtK-like protein with 75% of identity (4 amino acid substitutions) in the analyzed region. ^8^ The peptide SCS-α-12 also showed high identity levels with non-human pathogenic species of *Bartonella* genus, such as *Bartonella bovis*, *Bartonella senegalensis*, or *Candidatus* Bartonella rattimasiliensis, among others. ^9^ Strains: Heidi Mejia, Cond044, Hosp800-02, Peru38, VAB9028, and CAR600-02. ^10^ Strains: KC583, KC584, INS, San Pedro 600-02, Peru-18, Cusco-5, Ver-075, USM-LMMB 06, and USM-LMMB 07.

**Table 2:** Bacterial strains and plasmids used in the study.

	Description	Source or Reference
**Strains**		
*B. bacilliformis*	CIP 57.20—NCTC12136	Institute Pasteur
*E. coli* TOP10	Host strain for cloning	Invitrogen
*E. coli* BL21Star (DE3)	Host strain for gene expression	Invitrogen
**Plasmids**		
pCR4-TOPO	TA-cloning vector	Invitrogen
pET100D/TOPO	Expression vector	Invitrogen
pPap31	pET100D/TOPO containing Pap31	[5]
pPap31–A	pET100D/TOPO containing clone A of Pap31	This study
pPap31–B	pET100D/TOPO containing clone B of Pap31	This study
pPap31–C	pET100D/TOPO containing clone C of Pap31	This study
pSCS-α	pET100D/TOPO containing SCS-α	[5]
pSCS-α–A	pET100D/TOPO containing clone A of SCS-α	This study
pSCS- α–B	pET100D/TOPO containing clone B of SCS-α	This study
pSCS- α–C	pET100D/TOPO containing clone C of SCS-α	This study

SCS-α: succinyl-CoA synthetase subunit α.

**Table 3:** Primers used in the study.

	Sequence (5′→3′)	Fragment (bp)	Ref.
Pap31 F	^1^ CACCATGAATATAAAATGTTTAGTGACA	1–903	[5]
Pap31 R	TCAGAATTTGTAAGCAACACCAACGCG		
Pap31–A F	^1^ CACCATGAATATAAAATGTTTAGTGACA	1–325	This study
Pap31–A R	^2^ TCACAACCCAAACAATATCTG		
Pap31–B F	^1^ CACCATCTCGGCAGTGGCCTT	275–675	This study
Pap31–B R	^2^ TCATACCTGCTTTGCTAGCACTAC		
Pap31–C F	^1^ CACCTATCTTAAAAGGCACG	576–903	This study
Pap31–C R	TCAGAATTTGTAAGCAACACCAACGCG		
SCS α F	^1^ CACCATGTCAATTCTTATC	1–903	[5]
SCS α R	CTAACCCTTCAAGACTGAAACC		
SCS α–A F	^1^ CACCATGTCAATTCTTATC	1–381	This study
SCS α–A R	^2^ TCAACCAATTAAGCGCGATTTCG		
SCS α–B F	^1^ CACCTGTATTACAGAAGGTATACC	301–651	This study
SCS α–B R	^2^ TCAACCAATCTCACCGATC		
SCS α–C F	^1^ CACCGATGTGTTAGAAATGT	586–903	This study
SCS α–C R	CTAACCCTTCAAGACTGAAACC		

^1^ CACC at the 5′ end is a sequence added to allow pET directional cloning. ^2^ TCA at the 5′ end encodes for a STOP codon.

**Table 4:** Peptides used in the study.

Peptide		Peptide Sequence	Position (aa ^1^)
Pap 31	1	IGFGADRIMPYVSGGV	166–181
	2	GADRIMPYVSGGVAYT	169–184
	3	RIMPYVSGGVAYTQVQ	172–187
	4 ^2^	PYVSGGVAYTQVQAIG	175–190
	5	SGGVAYTQVQAIGSAI	178–193
	6	VAYTQVQAIGSAILKG	181–196
	7	TQVQAIGSAILKGTKD	184–199
	8	QAIGSAILKGTKDTGT	187–202
	9	GSAILKGTKDTGTEGG	190–205
	10	ILKGTKDTGTEGGGGT	193–208
	11	GTKDTGTEGGGGTEGG	196–211
	12	DTGTEGGGGTEGGGGT	199–214
	13	TEGGGGTEGGGGTEGG	202–217
	14	GGGTEGGGGTEGGGGS	205–220
	15	TEGGGGTEGGGGSASK	208–223
	16	GGGTEGGGGSASKAVR	211–226
	17	TEGGGGSASKAVRSEA	214–229
	18	GGGSASKAVRSEALDV	217–232
	19	SASKAVRSEALDVLAS	220–235
	20	KAVRSEALDVLASGTI	223–238
SCS-α	1	EQALAYHGTQMVGGIN	26–41
	2	LAYHGTQMVGGINPKK	29–44
	3	HGTQMVGGINPKKGGE	32–47
	4	QMVGGINPKKGGETWT	35–50
	5	GGINPKKGGETWTGAK	38–53
	6	NPKKGGETWTGAKGET	41–56
	7	KGGETWTGAKGETLPI	44–59
	8	ETWTGAKGETLPIFAS	47–62
	9	TGAKGETLPIFASVAE	50–65
	10	KGETLPIFASVAEGKE	53–68
	11	TLPIFASVAEGKEKTG	56–71
	12	IFASVAEGKEKTGANA	59–74
	13	SVAEGKEKTGANASVI	62–77
	14	EGKEKTGANASVIYVP	65–80
	15	EKTGANASVIYVPPAG	68–83
	16 ^2^	GANASVIYVPPAGAAD	71–86
	17	ASVIYVPPAGAADAII	74–89

^1^ Amino acid. ^2^ Pap31 peptide 4 and SCS-α peptide 16 were excluded from the study due to synthesis difficulties.

## Data Availability

The data presented in this study are available in [Immunogenic peptides from Pap31 and SCS-α of *Bartonella bacilliformis*: one step closer to a rapid diagnostic tool for Carrion’s disease].
